# 

**Published:** 1904-01-30

**Authors:** 


### Publications.

### CHARTS.

PRICES (Post or Carriage Paid):—

1,000, 25s.; 500, 13s. 6d.; 250, 7s. 6d.; 100, 3s. 6d.; 50, 2s.; 25, 1s. 3d.

SPECIMENS POST FREE.

THE BEST AND CHEAPEST PUBLISHED.

GOULD'S TEMPERATURE CHARTS, Morning and Evening.
GOULD'S FOUR-HOUR CHARTS, Printed in Violet for hours 2, 6, 16.
GOULD'S FOUR-HOUR CHARTS, Printed in Chocolate for hours 4, 8, 12.
GOULD'S TWO-HOUR CHARTS.
BENTON'S DIET CHARTS, each Chart lasting two days.
BOARDS to hold Charts when in use, 9d. each, 8s. 6d. per dozen.
PRIVATE NURSING CHARTS, lasting 48 hours.
CHARTS for taking the Temperature of Sick Rooms.

USED IN ALMOST ALL THE PRINCIPAL HOSPITALS AND INFIRMARIES IN THE UNITED KINGDOM.

**WODDERSPOON & CO., 5 Gate Street Lincoln's Inn Fields, W.C.**

---

Second Edition. Crown 8vo., price 3/6 net.

With original "Plea—1900—for the State Registration of Nurses."

### MOCK NURSES OF THE LATEST FASHION.

By FREDERICK J. GANT, F.R.C.S., Consulting Surgeon to the Royal Free Hospital.

BAILLIÈRE, TINDALL & COX, Henrietta Street, Covent Garden, London.

### THE HANDBOOK OF THE OPEN-AIR TREATMENT

By Dr. CHARLES REINHARDT

(Visiting Physician to Hailey Sanatorium, Wallingford, and Stourfield Park Sanatorium, Bournemouth),

Contains Views, Descriptions, and Information respecting 30 Sanatoria in Great Britain.

Price: Cloth, 2/6; Paper, 1/- (postage 4d.)

The "HEALTH RESORT" Publishing Office, 379 Strand, London, W.C.

---

Crown 8vo. Illustrated. Price 2s. 6d. net.

### ANÆSTHETICS. By J. BLUMFELD,

M.D.Cantab., Senior Anæsthetist to St. George's Hospital; Anæsthetist to the Grosvenor Hospital for Women and Children, London, and to the National Dental and Metropolitan Hospitals.

*Lancet.*—"....In writing this handbook the author has been quite successful." *Australasian Medical Gazette.*—"We can strongly commend this book." *Medical Chronicle.*—"The book is one which we can heartily recommend to senior students and practitioners."

London: BAILLIÈRE, TINDALL & COX, 8 Henrietta St., Covent Garden.

---

Crown 8vo. Illustrated. Price 1/- net.

### THE RADICAL CURE OF CORNS AND BUNIONS.

By E. HARDING FREELAND, F.R.C.S.

"Attention to the rules of treatment here suggested is likely to be the means of affording satisfaction to the practitioner and beneficial results to to the patient."—*Brit. Med. Jour.*

London: BALE, SONS & DANIELSSON, 83 Gt. Titchfield Street, W.

### DEATH.

NORMAN.—On January 21 at 27 Hyde Gardens, Eastbourne, Marian, third daughter of the late Charles Norman, after much suffering. (1319)

### NOTICE TO CORRESPONDENTS.

All MSS., Letters, Books for Review, and other matters intended for the Editor should be addressed THE EDITOR, "THE HOSPITAL," THE HOSPITAL BUILDINGS, 28 & 29 SOUTHAMPTON STREET, STRAND, W.C.

The Editor cannot undertake to return rejected MS., even when accompanied by stamped directed envelope.

**Notice to Advertisers and Subscribers.**

All Advertisements, Orders for Copies of THE HOSPITAL, and Business Communications should be addressed to **THE MANAGER (Not to the Editor),** THE HOSPITAL, 28 & 29 Southampton Street, Strand, London, W.C.

The Cost of Subscription, post free, is as follows:—Per week, 3½d.; per quarter, 3s. 9d.; per half-year, 7s. 6d.; per annum, 15s. Foreign and Colonial:—Per annum, 19s. 6d., payable, in all cases, in advance.

**NOTICE.—Vol. XXXIV. of "The Hospital" (April 1903 to September 1903), in handsome cloth binding, is now on sale at the office of this Journal, price 10s. 6d. Cases for binding the Numbers contained in this Volume 2s. Index to Vol. XXXIV., 2½d., post free.**

**The Monthly part for February will be ready on Feb. 1. Price 1s., by post, 1s. 3d.**

### BOOKS RECEIVED.

LOCAL GOVERNMENT JOURNAL OFFICE.

"Government Official Directory." By S. E. Rogers.

SCIENTIFIC PRESS, LIMITED.

"Scheme for the Provision of Nursing Homes at Moderate Prices."

WALTER SCOTT PUBLISHING CO.

"Business Success." By G. G. Millar.

R. B. JOHNSON,

"The Food for the Gods." By B. Head.

GRANT RICHARDS.

"How to Keep Well." By F. Crandall.

### THE HOSPITAL LIBRARY AND CHARITIES BUREAU.

Inquiries of the librarian are answered in this column free of charge. Inquiries to be answered promptly by post must be accompanied by a fee of 2s. 6d.

*Classification of Expenditure.*

What items of hospital expenditure should be classified as extraordinary expenditure.

"The Uniform System of Accounts," by Sir Henry Burdett (The Scientific Press, 28 Southampton Street), gives full directions for the proper keeping of hospital accounts and a list of items which should be classified under extraordinary expenditure.

*The Hospital Commissariat.*

Is it possible to ascertain what is the amount paid for provisions at the large London hospitals, and further, under the various items of bread, meat, groceries, etc.? Do the various hospitals publish balance-sheets which are accessible to the ordinary public?

The total amount paid for provisions is stated in the annual reports of the hospitals, but few publish the separate items. Balance-sheets are to be found in these reports.

### THE STUDENT OF MEDICINE.

### APPOINTMENTS.

**H. W. Beach**, M.R.C.S., L.R.C.P., Medical Officer of Health to the Yarmouth Town Council. **H. Buckland-Jones**, M.B., M.Ch.Edin., Assistant Surgeon to the Metropolitan Ear, Nose, and Throat Hospital. **A. Coleridge**, M.R.C.S., L.R.C.P., Senior Resident Medical Officer at the Mount Vernon Consumption Hospital, Hampstead, N.W. **A. P. Condell**, M.D.Manitoba, Clinical Assistant to the Chelsea Hospital for Women. **C W. Donald**, M.B., F.R.C.S.Edin., Assistant Honorary Physician to the Cumberland Infirmary, Carlisle. **Sidney R. Dyer**, M.D.Brux., M.R.C.S.Eng., L.R.C.P.Lond., D.P.H.Eng., Barrister-at-Law of the Middle Temple, Medical Officer, H.M. Prison, Dartmoor. **J. Howell Evans**, M.A., M.B., M.Ch., F.R.C.S., Registrar to the Chelsea Hospital for Women. **C. W. Forsyth**, M.R.C.S., L.R.C.P., Resident Assistant Medical Officer of the Workhouse of the South Shields Union. **Robert Goodwin**, M.D., C.M.Manitoba, Clinical Assistant to the Chelsea Hospital for Women. **Lachlan Grant**, M.D., C.M.Edin., Medical Officer to the Ballachulish Quarries Medical Club. **H. Grey-Edwards**, M.D., M.Ch.Dub., Honorary Ophthalmic Surgeon to the Carnarvonshire and Anglesey Infirmary, Bangor. **H. Hine**, M.B., F.R.C.S., Medical Officer of the Infirmary and the Newark District of the Newark Union. **Sydney Jamieson**, M.B., Ch.M.Edin., Lecturer on Medical Jurisprudence at the University of Sydney. **A. W. Jenkins**, M.B.Lond., M.R.C.S., District Medical Officer of the Hinckley Union. **R. H. Jones**, M.B., Ch.M.Melb., Honorary Assistant Ophthalmic Surgeon at the Sydney Hospital. **W. T. Lister**, B.A., M.B., B.C., F.R.C.S., Assistant Surgeon to the Royal London Ophthalmic Hospital, City Road, E.C. **A. C. Miller**, M.D.Edin., Medical Officer to the Post Office for the Fortwilliam District. **R. E. Nix**, M.B., B.C.Cantab., Medical Officer of Health to the Chatteris Urban District Council. **E. O. Price**, M.D.Edin., Honorary Medical Officer to the Carnarvonshire and Anglesey Infirmary, Bangor. **Thomas Rose**, M.R.C.S, L.R.C.P., Resident Medical Officer to the Chelsea Hospital for Women. **Alec Waugh**, M.B., C.M.Glasg., Medical Officer, and Public Vaccinator, Skipton District, Skipton Union. **G. W. Weir**, M.D., M.Ch., R.U.I., D.P.H.Camb., Medical Officer of the Workhouse of the South Shields Union.

DERBYSHIRE ROYAL INFIRMARY.—The following appointments have been made:—House Surgeon: **Basil M. Wilson**, M.B Vict. House Physician: **Rupert Butterworth**, M.B.Cantab. House Surgeon No. 2: **Ernest Bennett**, M.B.Glasgow. Assistant House Surgeon: **Percy Hardy**, M.R.C.S., L.R.C.P.

**"The Hospital" Institutional Library.**

"Hospitals and Asylums of the World." 4 Vols., with a Portfolio of Plans, complete £12 12s.

"Burdett's Hospitals and Charities, 1903." 5s. net.

"Burdett's Official Nursing Directory, 1903." 3s. net.

"Cottage Hospitals, General, Fever, and Convalescent." 10s. 6d.

"Uniform System of Accounts for Hospitals and Public Institutions." New Edition. 4s. net.

"Hospital Expenditure: The Commissariat." 2s. 6d.

"A Legal Handbook for the Use of Hospital Authorities." 2s. 6d.

"The Cottage Hospital Case Book or Register of Patients." 10s. and 12s. 6d.

All these are published by the SCIENTIFIC PRESS, Ltd., and may be obtained through any bookseller, or direct from the publishers, 28 and 29 Southampton Street, Strand, London, W.C.

**STERILIZATION OF CERTAIN DEGENERATES.**

*Appeal to Asylum Managers.*

**By ROBERT R. RENTOUL, M.D.**

Price 1s. net; by post, 1s. 1½d.

THROUGH ANY BOOKSELLER.

### THE NURSING PROFESSION: How and Where to Train.

**By Sir HENRY BURDETT, K.C.B.**

**A reliable and up-to-date Guide to Training for the calling of a Nurse. In its pages would-be Nurses will find all the information they require.**

New Edition. Price **2/-**, post free **2/4.**

**Of all Booksellers or of** THE SCIENTIFIC PRESS, LTD., **28 & 29 Southampton Street, Strand, London, W.C.**

### Two Useful Text-books.

**ELEMENTARY ANATOMY AND SURGERY FOR NURSES.**

By WILLIAM McADAM ECCLES, M.S. Lond., M.B., F.R.C.S., L.R.C.P.

Crown 8vo., profusely illustrated, cloth, **2/6** post free.

"The book will be valuable to nurses who are beginning the study of anatomy. The instruction is given very clearly and concisely, and the reader is able to glean much information in a very condensed form."—*Nursing Notes.*

**ELEMENTARY PHYSIOLOGY FOR NURSES.**

By C. F. MARSHALL, M.D., B.Sc., F.R.C.S.

Crown 8vo., illustrated, cloth, 2/- post free.

"Nurses will find in it all that they require to know of the subjects treated."—*Daily Chronicle.*

*To be obtained of all Booksellers, or of*

THE SCIENTIFIC PRESS, LTD., 28 & 29 Southampton Street, Strand, London, W.C.

*Just Published.*

### THE EXAMINATION OF THE URINE.

By J. K. WATSON, M.D.Edin., M.B., C.M.

*Author of "A Handbook for Nurses."*

Price **1/-** net; post free **1/1.**

*Of all Booksellers, or of*
THE SCIENTIFIC PRESS, LTD, 28 & 29 Southampton Street, Strand, London, W.C.

### For District Nurses and Midwives.

### PRACTICAL HINTS ON DISTRICT NURSING.

By AMY HUGHES,

Late of the Central Training Home, Queen Victoria's Jubilee Institute for Nurses for the Sick Poor. Price **1/-** post free.

### THE ADVANTAGES AND PRIVILEGES OF A TRAINED DISTRICT NURSE AND THE DUTIES OF THE DISTRICT TOWARDS HER.

By SIR HENRY BURDETT, K.C.B.

An interesting little pamphlet. Price **2d.** post free.

### THE POCKET CASE BOOK FOR DISTRICT AND PRIVATE NURSES.

Suitable for Pocket. Cloth boards, price **1/-**; leather, **1/6** post free.

### ON PREPARATION FOR OPERATION IN PRIVATE HOUSES.

By A SURGEON.

Price **6d.** post free.

### A PRACTICAL HANDBOOK OF MIDWIFERY.

By FRANCIS HAULTAIN, M.D.

An invaluable complete and advanced handbook for practising midwives.

Bound in leather, **6/3** post free.

### THE MIDWIVES' POCKET BOOK.

With Chapter on the Midwives Bill.

By HONNOR MORTEN.

A valuable little book. Price **1/-** post free.

### THE ART OF FEEDING THE INVALID.

By A MEDICAL PRACTITIONER AND A LADY PROFESSOR OF COOKERY.

Price **1/6** post free.

### THE PHYSIOLOGICAL FEEDING OF INFANTS.

By ERIC PRITCHARD, M.D.

A scientific guide to the artificial feeding of infants from birth. Price **1/-** post free.

*These books are obtainable of all Booksellers and are published by*
THE SCIENTIFIC PRESS, LTD., 28 and 29 Southampton Street, Strand, London, W.C.,
*who will send their latest Catalogue of Nursing Manuals, post free, to any Nurse on application.*

